# Limited effects from professional identity formation-oriented intervention on self-regulated learning in a preclinical setting: a randomized-controlled study in Japan

**DOI:** 10.1186/s12909-020-02460-3

**Published:** 2021-01-07

**Authors:** Yasushi Matsuyama, Motoyuki Nakaya, Jimmie Leppink, Cees van der Vleuten, Yoshikazu Asada, Adam Jon Lebowitz, Teppei Sasahara, Yu Yamamoto, Masami Matsumura, Akira Gomi, Shizukiyo Ishikawa, Hitoaki Okazaki

**Affiliations:** 1 Medical Education Center, Jichi Medical University, 3311-1 Yakushiji, Shimotsuke, Tochigi Japan; 2 The department of Psychology and Human Developmental Sciences, Nagoya University, Furo-cho, Chikusa-ku, Nagoya, Japan; 3 Hull York Medical School, University of York, Heslington, York, UK; 4 Department of Educational Development and Research, Faculty of Health, Medicine, and Life Sciences, Maastricht University, 6200 MD Maastricht, The Netherlands; 5 Center for Information, Jichi Medical University, 3311-1 Yakushiji, Shimotsuke, Tochigi Japan; 6 Department of General Education, Jichi Medical University, 3311-1 Yakushiji, Shimotsuke, Tochigi Japan; 7 Department of Infection and Immunity, Jichi Medical University, 3311-1 Yakushiji, Shimotsuke, Tochigi Japan; 8 Division of General Medicine, Center for Community Medicine, Jichi Medical University, 3311-1 Yakushiji, Shimotsuke, Tochigi Japan; 9 Department of Pediatric Neurosurgery, Jichi Children’s Medical Center Tochigi, Jichi Medical University, 3311-1 Yakushiji, Shimotsuke, Tochigi Japan

**Keywords:** Self-regulated learning, Professional identity formation, Problem-based learning, Teacher-centered learning, Learning management system

## Abstract

**Background:**

Developing self-regulated learning in preclinical settings is important for future lifelong learning. Previous studies indicate professional identity formation, i.e., formation of self-identity with internalized values and norms of professionalism, might promote self-regulated learning. We designed a professional identity formation-oriented reflection and learning plan format, then tested effectiveness on raising self-regulated learning in a preclinical year curriculum.

**Methods:**

A randomized controlled crossover trial was conducted using 112 students at Jichi Medical University. In six one-day problem-based learning sessions in a 7-month pre-clinical year curriculum, Groups A (*n* = 56, female 18, mean age 21.5y ± 0.7) and B (*n* = 56, female 11, mean age 21.7y ± 1.0) experienced professional identity formation-oriented format: Group A had three sessions with the intervention format in the first half, B in the second half. Between-group identity stages and self-regulated learning levels were compared using professional identity essays and the Motivated Strategies for Learning Questionnaire.

**Results:**

Two-level regression analyses showed no improvement in questionnaire categories but moderate improvement of professional identity stages over time (*R*^2^ = 0.069), regardless of timing of intervention.

**Conclusions:**

Professional identity moderately forms during the pre-clinical year curriculum. However, neither identity nor self-regulated learning is raised significantly by limited intervention.

## Background

Self-regulated learning (SRL) is defined as learners’ active participation in learning process from metacognitive, motivational, and behavioral perspectives [1]. Medical professionals are required to update knowledge autonomously in line with rapid advances in medicine, and self-regulation in clinical contexts has become an essential competency for medical professionals [2–5]. Zimmerman described SRL as a cyclical process in three phases [6, 7].

1. *Forethought phase*: Learners set goals and choose strategies to attain them
2. *Performance phase*: Learners monitor and control behavior to attain goals
3. *Self-reflection phase*: Learners formulate new goals and strategies for future situations by reflecting on the previous study performance

Because present preclinical education cannot prepare learners for all challenges faced in less-structured learning situations in clinical settings, robust development of SRL-oriented education in preclinical settings is justified [5, 8–10].

### SRL-oriented interventions in preclinical settings

SRL-oriented educational interventions in preclinical settings include two main approches. The first approach is microanalysis. It is aimed at individualized assessment and modification for learning-specific processes in a specific learning context (e.g., clinical reasoning) [11, 12]. This approach may allow preclinical year students to acquire specific SRL skills for specific learning tasks performed in the later clinical clerkship. However, it is also important to have students acquire SRL skills that can be applied to a wider variety of learning tasks in the clinical clerkship. From this perspective, another approach is to implement SRL-oriented intervention in preclinical education, with reference to reports of interventions that have improved SRL in clinical practice.

A systematic review by van Houten-Schat and colleagues [8] summarized educational intervention studies for SRL in clinical settings by categorizing three areas: (1) SRL guidance via mentoring, (2) goal setting and learning plan support, and (3) online support tools. Studies have confirmed incorporation of these components into medical education programs has potential positive impacts. For example, Stuart et al. [13] found that guidance on the use of strategies and learning plans for recording goal achievement raised students’ awareness of the learning process.

Other reports indicate that educational interventions could focus on professional identity formation (PIF), another contextual attribute that might foster SRL in clinical settings [4, 14–22]. According to Cruess et al. ([14], p1447), professional identity is defined as “a representation of self, achieved in stages over time during which the characteristics, values, and norms of the medical profession are internalized”. One study has shown when physicians perceive their identity as professionals, they begin to view daily learning tasks as high-stakes, and to self-regulate learning behaviours as coping strategies [16]. Other studies have shown that explicit future professional self-imaging during clinical clerkship leads to self-reflection, increased attention to learning strategies of professional role models, and diversification of learning strategies [17, 18].

In response to advances in medical science and increasingly diverse needs of society, “professional” attributes such as *autonomy*, *self-regulation*, and *social responsibility* have been emphasized, in addition to traditional moral and ethical education emphasizing *healer* roles [19, 20]. Rather than impart knowledge of professionalism superficially, educating medical students to internalize the values and norms of the healthcare profession has been emphasized. During the formation of professional identity, medical students begin to perceive belonging to a professional community, and to pay increased attention to and emulate the behaviours of role models [21–23]. Therefore, students’ self-reflection by comparison with the learning behaviours of role models can be promoted. When attention to and emulation of behaviours of role models increases, students will also be likely to adopt the learning strategies used by those role models. As professional identity continues to develop, students gain the ability to think about themselves in relation to the larger systems within their professional communities [21–23].

Moreover, people are more likely to accept difficult experiences by implying task importance when an accessible identity feels congruent with the task [24, 25]. In the context of this study, the growing professional identity of ‘physician-to-be’ might strengthen perception of the importance of engaging in challenges during preclinical learning and in self-regulating one’s learning behaviours using learning beliefs consistent with professional identity. Given that PIF is associated with self-reflection, diversified learning strategies and motivational states, SRL may be facilitated by introduction of a PIF-oriented intervention.

To date, the question of whether educational interventions in addition to regular instruction establish SRL in pre-clinical settings has not been fully explored. While the robust development of SRL-oriented education in preclinical settings has been emphasized, it is problematic that no actual intervention studies have been conducted using SRL as an outcome for students prior to clinical clerkships.

### PIF-oriented intervention for preclinical year curriculum

This study specifically focused on PIF as a facilitating factor for SRL. This focus was based on previous studies [16–18] showing the possible benefits of PIF-oriented intervention even for East Asian medical students, who are considered less amenable to SRL because of the influence of pre-university education, with its strong faculty instruction and in-university, teacher-centred curriculum.

**Fig. 1:**
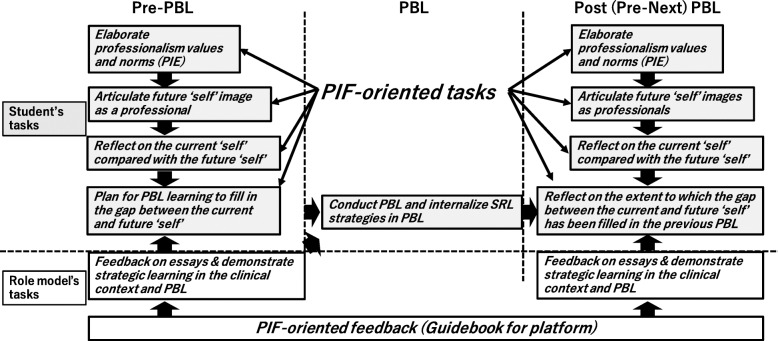
Conceptual framework of professional identity formation-oriented pre-PBL intervention. PIE: professional identity essay, PIF: professional identity formation, SRL: self-regulated learning

In this study, we applied Matsuyama et al.’s PIF-oriented reflection and learning plan platform [18] (Fig. 1) in the preclinical year curriculum and used six one-day PBL sessions as intervention points in the preclinical year seven-month research period. Characteristics of the platform are summarized as follows:

1. reflecting on the current “self” compared with the future “self” while articulating values/norms of professionalism by professional identity essay [22] and imaging the future “self” with the professionalism values/norms
2. strategic learning plan for an upcoming learning opportunity to fill in the gap between the current and future “self” while students adopt learning strategies suggested by role models

We used one-day PBL sessions as intervention points because PBL originally included elements of planning and self-reflection for self-study [26, 27] and seemed to be effective in setting up a control group for comparison with the intervention group. That is, students in the intervention group could implement self-reflection and learning plans by referring to their future professional self-image, reinforced by feedback from their role models. On the other hand, the control group implemented a learning plan and self-reflection embedded in the structure of PBL without reinforcement of professional identity. This difference was expected to make the effects of the PIF-oriented intervention more apparent.

The purpose of this study was to examine whether a PIF-oriented intervention would enhance SRL among preclinical year students. The following research questions were formulated.

- Research Question 1: Does a professional-identity formation oriented reflection and learning plan platform improve SRL levels in a preclinical year curriculum?
- Research Question 2: Does SRL level improve in parallel with professional-identity formation development?

This study was approved by the university’s ethics committee (reference number: 18–168). Informed consent was obtained from all participants. This study was conducted from May 2019 to April 2020.

## Methods

### Settings

Jichi Medical University (JMU) is a private medical university founded in 1972 in Japan. The curriculum of JMU complies with the standardized model core curriculum outlining fundamental learning contents for undergraduate medical education in Japan [28]. While the preclinical curriculum is partly integrated, it remains mostly stepwise. In JMU, students mainly learn clinical medicine in traditional didactic lectures before Year 4 to 6 (Y4–6) clinical clerkship. JMU students study liberal arts in the first and second trimesters of Y1. Lectures and experiments in basic medicine also begin in Y1 second trimester. Clinical medicine lectures start from Y2 second trimester. Before Y3 end, students finish almost all subject lectures in basic and clinical medicine. Each basic and clinical medicine class is capped by end-of-unit tests where lecturers create test items based on lecture content.

Y3 has seven sessions of one-day hybrid problem-based learning (PBL), each divided into four segments: morning case discussion for the formulation of self-study objectives, self-study period for research on objectives and preparation for afternoon discussion, afternoon discussion including within-group information sharing, and a 60-min wrap-up lecture from a specialist.

Currently, students read the introductory part of a clinical scenario before PBL and preview morning discussion content. For example:

‘A 56-year-old man came to your hospital because he had 20-min anterior chest oppression after breakfast this morning. Please find clinical problems or possible differential diagnoses as best you can.’

The full story containing clinical history and findings is provided on the date of the PBL session.

### PIF-oriented intervention platform

The core component of the PIF-oriented platform is composed of an online instruction video and an essay format. The instruction video aimed at encouraging students to articulate their future image as an independent medical professional tackling patient problems via life-long learning. The essay format meant to provide pre-PBL in-depth communication between students and their role models (Fig. 1).

After watching the instruction video and reading the PBL scenario introductory, students were asked to answer the following essay questions:

- *Q1: Please formulate your future professional images, and articulate how useful this PBL case-study would be to you as a doctor responsible for this case.*
- *Q2. Based on your answer for Q1, please articulate how you will optimize your self-study for this case to make this opportunity most meaningful.*

Aside from these questions, students were asked to submit the professional identity essay (PIE) [22] three times during the research period. PIE is useful for helping learners articulate their values and norms about medical professionalism, and role model doctors provide feedback by referring to rubrics based on Kegan’s constructive developmental theory [21–23]. This study used a Japanese version of PIE (PIE-J) as a reference for role models’ feedback on Q1 and 2 and as an assessment tool for students’ professionalism levels (see also *Instruments*). The professional identity essay-based feedback was also aimed at the remediation of self-images with underdeveloped professionalism (low developmental stages in PIE) in the intervention group.

Those materials were provided on a Moodle online learning management system. Through the Moodle platform, eligible JMU-graduate doctors provided feedback on Q1 and Q2 by simultaneously referring to each student’s PIE. As a rule, the feedback did not contain hints for the PBL scenario in order to avoid teacher-centered instruction. In this study, six JMU graduates with clinical experience of 18–37 years were chosen as role models and feedback providers. Students were allowed to continue communicating with their role models via the Moodle platform until they were satisfied. Before the study, all role models received intensive training for appropriate PIE use and feedback on Q1 and 2.

We hypothesized the online pre-PBL platform would raise student awareness of their future professional image and relevance of PBL scenarios for their future professional selves. We also proposed that a clearer image of their future professional selves and relevance of PBL contents would encourage preclinical year students to acquire learning strategies encouraged by thoughtful role models’ comments. Overall, we expected that the pre-PBL PIF-oriented platform would promote PIF and SRL in a parallel manner.

### Participants and design

**Fig. 2:**
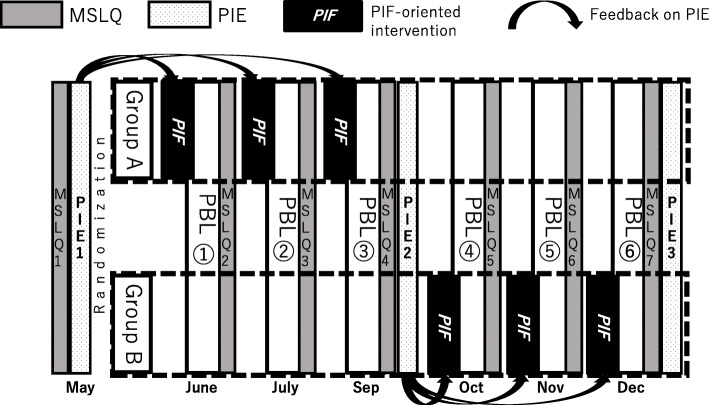
Overview of the randomized controlled crossover trial

A randomized controlled trial was designed. All JMU 2019 Y3 students (*n* = 124) were invited to participate in this research. Eventually, 112 agreed were randomly divided into two groups: Group A (*n* = 56, female = 18, male = 38, mean age 21.5y ± 0.7) and Group B (*n* = 56, female = 11, male = 45, mean age 21.7y ± 1.0). Group A used Moodle-based PIF-oriented platform before the second to fourth PBL, while group B did before the fifth to seventh PBLs in 2019. Both groups conducted the six one-day PBLs in the same manner on PBL dates, and SRL and PIF levels were compared between the two groups (Fig. 2). Group A and B did not mix in the PBL group session.

We conducted the randomized controlled trial in a crossover manner to ensure all students had the same overall learning experience [29], even though the effects of the PIF-oriented intervention had remained unclear.

We hypothesized that PIF and SRL levels should improve in parallel, i.e., Group A in the first half of the research period, with Group B becoming equivalent to Group A in the second half.

### Instruments

#### PIF data collection

The PIF levels for norms and values of professionalism were measured using PIE, an essay-based measurement tool with 9 question items. Referring to Dr. Kalet and colleagues’ rubric based on Kegan’s identity stage [22], assessors chose learners’ professional identity levels from Stage II to II/III, III, III/IV, IV, IV/V, and V. The PIF measurement by PIE has been validated in undergraduate settings [22, 30]. In this study, we used a Japanese version of the PIE form and rubric (PIE-J) originally in English. Back translation between English and Japanese was conducted by the main author (YM, Japanese) and an American professor living in Japan literate in both English and Japanese (AJL). PIE stages from all students were assessed by two authors (YM & MN) by in-depth discussions following the rubric until full agreement was reached.

#### SRL data collection

Learners’ SRL levels were measured by a Japanese-language version of the Motivated Strategies for Learning Questionnaire (MSLQ-J) [31] reported to be useful in measuring SRL in undergraduate medical education [18, 32, 33]. MSLQ is composed of 81 items with seven-point Likert scales which quantify levels of nine types of learning strategies (rehearsal: R, elaboration: ELA, organization: O, critical thinking: CT, metacognitive self-regulation: MSR, time and study environment: TaSE, effort regulation: ER, peer learning: PL, and help-seeking: HS), and six variables of motivation states (intrinsic goal orientation: IGO, extrinsic goal orientation: EGO, task value: TV, control of learning beliefs: CBaL, self-efficacy for learning and performance: SEfLaP, and test anxiety: TA). All 81 items were translated into Japanese and back-translated by the main author (YM) and an American professor (AJL).

### Analysis

Effects of treatment and time (fixed effects) on MSLQ-J scores and PIE-J stages were tested and estimated using two-level regression analysis (upper level: participants; lower level: occasion) in the Open Source statistical package *jamovi* (version 1.2.9) [34]. Treatment and time were treated as fixed effects (estimated with full informed maximum likelihood), and participant-level random intercept served as a random effect (estimated with restricted maximum likelihood). For the first measurement of all scales, the two groups were treated as one because the first measurement took place before any treatment (see Chapter 15 in [35] for a detailed explanation of this model and the rationale behind it). Marginal *R*^2^, a multilevel equivalent of the *R*^2^-statistic commonly used for traditional linear regression models, was used to estimate the effects of time and treatment (values of around 0.01, 0.06 and 0.14 represented small, medium and large effects). The Bayesian Information Criterion (BIC) was used to determine which of the time-effect-only and the time-and-treatment-effect model is to be preferred (i.e., the model with the smallest BIC) [35]. Correlations between MSLQ-J scores and PIF-J stages were analyzed and visualized using network analysis in the Open Source statistical package JASP (version 0.12.1.0) [36].

## Results

Table 1 shows the mean ± SD of 15 MSLQ-J categories in Group A and Group B and the distribution of PIE-J stages.

**Table 1:** (a) The mean ± SD values of 15 MSLQ-J categories. (b) Distribution of PIE-J stages in Group A and Group B

(a)								
MSLQ	Group	Baseline	PBL1	PBL2	PBL3	PBL4	PBL5	PBL6
IGO	A	3.76 ± 1.05	3.84 ± 1.27	3.90 ± 1.29	4.15 ± 1.35	4.04 ± 1.49	4.17 ± 1.46	4.12 ± 0.99
	B	4.10 ± 1.25	4.23 ± 1.00	4.12 ± 1.33	4.15 ± 1.17	4.22 ± 1.27	4.34 ± 1.64	4.34 ± 1.25
EGO	A	3.34 ± 1.29	3.48 ± 1.33	3.63 ± 1.43	3.75 ± 1.43	3.66 ± 1.56	3.54 ± 1.53	3.84 ± 1.38
	B	3.68 ± 1.32	3.69 ± 1.15	3.82 ± 1.34	3.94 ± 1.30	3.91 ± 1.41	3.94 ± 1.62	3.81 ± 1.43
TV	A	4.99 ± 0.91	4.64 ± 1.41	4.48 ± 1.38	4.74 ± 1.54	4.59 ± 1.58	4.56 ± 1.65	4.74 ± 1.10
	B	4.95 ± 0.97	4.91 ± 0.93	4.49 ± 1.29	4.74 ± 1.33	4.64 ± 1.44	4.59 ± 1.77	4.90 ± 1.31
CBaL	A	4.57 ± 0.93	4.41 ± 1.21	4.21 ± 1.18	4.33 ± 1.32	4.21 ± 1.44	4.22 ± 1.53	4.47 ± 1.00
	B	4.63 ± 0.99	4.64 ± 0.83	4.27 ± 1.27	4.43 ± 1.21	4.41 ± 1.30	4.37 ± 1.59	4.60 ± 1.20
SEfLaP	A	3.00 ± 1.19	3.42 ± 1.37	3.51 ± 1.26	3.64 ± 1.34	3.66 ± 1.46	3.92 ± 1.56	3.75 ± 1.17
	B	3.24 ± 1.16	3.68 ± 0.96	3.90 ± 1.33	4.07 ± 1.16	4.03 ± 1.26	4.03 ± 1.50	3.94 ± 1.19
TA	A	4.09 ± 1.24	4.06 ± 1.28	4.10 ± 1.43	4.15 ± 1.41	4.10 ± 1.60	3.94 ± 1.59	4.13 ± 1.30
	B	3.79 ± 1.26	3.90 ± 1.05	3.83 ± 1.28	4.03 ± 1.15	3.75 ± 1.30	3.83 ± 1.51	3.99 ± 1.27
R	A	3.93 ± 0.90	4.19 ± 1.20	4.20 ± 1.22	4.19 ± 1.32	4.10 ± 1.43	4.17 ± 1.45	4.21 ± 1.03
	B	3.79 ± 1.12	4.13 ± 0.86	4.15 ± 1.25	4.05 ± 1.16	4.12 ± 1.21	4.42 ± 1.64	4.37 ± 1.14
ELA	A	4.72 ± 0.90	4.47 ± 1.19	4.39 ± 1.20	4.55 ± 1.40	4.48 ± 1.54	4.41 ± 1.56	4.57 ± 1.06
	B	4.59 ± 1.14	4.54 ± 0.90	4.44 ± 1.33	4.40 ± 1.22	4.35 ± 1.38	4.38 ± 1.66	4.60 ± 1.22
O	A	4.18 ± 1.26	4.31 ± 1.34	4.34 ± 1.37	4.46 ± 1.51	4.42 ± 1.57	4.41 ± 1.60	4.40 ± 1.14
	B	4.09 ± 1.14	4.21 ± 1.02	4.38 ± 1.32	4.33 ± 1.34	4.20 ± 1.35	4.22 ± 1.61	4.44 ± 1.28
CT	A	4.15 ± 0.85	4.28 ± 1.14	4.29 ± 1.27	4.18 ± 1.32	4.20 ± 1.51	4.22 ± 1.47	4.26 ± 0.88
	B	4.14 ± 1.08	4.20 ± 0.76	4.22 ± 1.27	4.09 ± 1.23	4.12 ± 1.22	4.14 ± 1.57	4.36 ± 1.15
MSR	A	4.06 ± 0.62	4.22 ± 0.99	4.26 ± 1.06	4.24 ± 1.17	4.25 ± 1.31	4.27 ± 1.34	4.28 ± 0.65
	B	4.03 ± 0.77	4.19 ± 0.55	4.16 ± 1.12	4.16 ± 0.95	4.15 ± 1.12	4.20 ± 1.43	4.33 ± 0.98
TaSE	A	4.25 ± 0.74	4.23 ± 0.99	4.21 ± 1.00	4.46 ± 1.18	4.28 ± 1.28	4.29 ± 1.36	4.37 ± 0.63
	B	4.15 ± 0.77	4.28 ± 0.66	4.32 ± 1.13	4.22 ± 0.98	4.06 ± 1.12	4.33 ± 1.46	4.38 ± 1.01
ER	A	4.50 ± 0.89	4.46 ± 1.17	4.21 ± 1.07	4.38 ± 1.20	4.31 ± 1.35	4.32 ± 1.41	4.31 ± 0.63
	B	4.28 ± 0.91	4.49 ± 0.81	4.26 ± 1.22	4.21 ± 1.11	4.14 ± 1.20	4.31 ± 1.51	4.29 ± 1.10
PL	A	4.51 ± 1.05	4.33 ± 1.38	4.27 ± 1.32	4.44 ± 1.53	4.33 ± 1.60	4.36 ± 1.65	4.40 ± 1.09
	B	4.56 ± 1.27	4.64 ± 0.94	4.38 ± 1.42	4.37 ± 1.32	4.30 ± 1.43	4.25 ± 1.73	4.50 ± 1.32
HS	A	4.46 ± 0.93	4.36 ± 1.23	4.23 ± 1.16	4.32 ± 1.25	4.25 ± 1.40	4.34 ± 1.54	4.29 ± 0.82
	B	4.58 ± 0.77	4.42 ± 0.71	4.25 ± 1.21	4.21 ± 1.16	4.18 ± 1.21	4.39 ± 1.53	4.31 ± 1.10
(b)								
PIE-J stagesI	Group A			Group B				
	PIE1	PIE2	PIE3	PIE1	PIE2	PIE3		
II	7	2	2	6	5	1		
II/III	21	14	15	28	18	15		
III	22	20	10	16	26	18		
III/IV	4	13	19	4	3	13		
IV	0	0	8	0	1	3		
IV/V	0	0	0	0	0	0		
V	0	0	0	0	0	0		

In Table 2, a two-level regression analysis showed the *R*^2^-value of 0.069 in the time-effect-only model in PIE indicated moderate improvement of PIF-J stages over time. However, the time-effect-only model and the time-and-treatment-effect model yield almost the same marginal *R*^2^-value and BIC. Therefore, there is no reason to assume treatment effects beyond time-effects only. For the 15 MSLQ scales, there were relatively small differences in score between times (i.e., marginal *R*^2^-values in the 0.002–0.034 range) which lead to the same conclusion about treatment: almost no change in marginal *R*^2^-value and BIC due to treatment over the time-effect-only model.

**Table 2:** Two-level regression with participant-level random intercept and fixed effects of time and treatment for PIE and the 15 MSLQ scales

	Random	Fixed part			
Scale	Intercept	Time and treatment effect		Time effect only	
	ICC	***R***^**2**^	BIC	***R***^**2**^	BIC
**PIE**	0.66	0.07	410.143	0.069	399.323
**MSLQ**					
IGO	0.269	0.007	10070.636	0.005	10027.532
EGO	0.346	0.007	10420.826	0.005	10377.412
TV	0.312	0.014	14179.596	0.012	14136.058
CBaL	0.221	0.011	9878.656	0.009	9832.988
SEfLaP	0.334	0.036	19428.128	0.034	19388.875
TA	0.253	0.002	13404.573	0.002	13357.443
R	0.165	0.01	10571.942	0.008	10529.513
ELA	0.292	0.006	14222	0.005	14178.355
O	0.319	0.005	9866.504	0.004	9822.927
CT	0.263	0.003	11751.478	0.002	11703.7
MSR	0.122	0.003	29923.339	0.003	29872.557
TaSE	0.085	0.004	22008.123	0.002	21966.763
ER	0.157	0.005	10085.647	0.004	10041.528
PL	0.296	0.006	7569.559	0.004	7529.637
HS	0.112	0.004	10977.313	0.004	10930.299

Intraclass coefficient (ICC), marginal *R*2-value,and Bayesian information criterion (BIC)

**Fig. 3:**
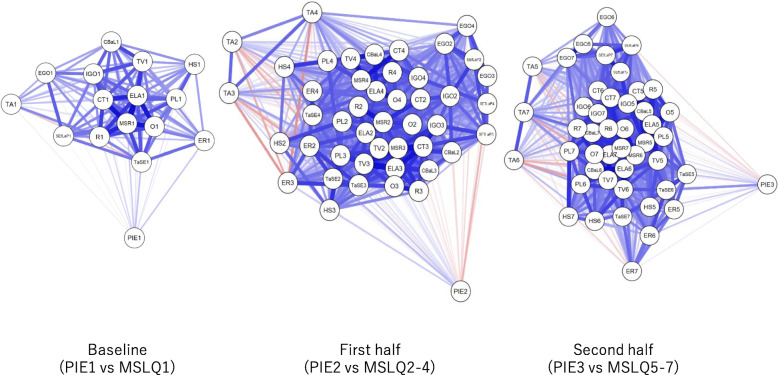
Network plot of PIE and MSLQ categories. Blue and red lines represent positive and negative correlations, respectively. Thicker lines represent stronger correlations. Weaker lines usually result in longer lines to indicate which variables are more closely related

Figure 3 shows the network plot of PIE-J stages and MSLQ-J category scores in the three phases, with thicker lines reflecting stronger correlations than thinner lines, and blue for positive/red for negative correlations. Overall, correlations between PIE and MSLQ-J scales were weak (PIE1*MSLQ1: − 0.066 to 0.186; PIE2*MSLQ2–4: − 0.244 to 0.220; PIE3*MSLQ: 5–7: − 0.140 to 0.381).

## Discussion

Contrary to the hypothesis, PIF-oriented intervention in this study did not significantly improve PIF or SRL. Moreover, contrary to the previous study results in clinical contexts [17, 18], the PIF improvement did not contribute to the improvement in SRL. This study did not show any significant results in terms of testing the hypothesis that PIF-oriented intervention improves students’ SRL in a preclinical year curriculum. However, a few important findings were noted.

First, PIF stages among the students participating in this study were low. Although not directly comparable, according to a study of 132 entering U.S. medical school students [22] and a study comparing 122 students at admission and preclinical clearkship training [30], none of the students were at Stage II. Plus, only a few students in the latter study [30] were judged to be at Stage II/III. Most students in the both studies were at Stage III or III/IV [22, 30]. On the other hand, one-third to one-half of the students in this study were at Stage II/III and a few were at Stage II (Table 1b), although they had studied more than 2 years since admission and were now within 1 year of commencing the clinical clerkship. Due to the difference in the admission system to medical school, U.S. medical students enter medical school after graduating from college after potentially completing a different major [37], whereas Japanese medical students enter medical school directly from high school age 18 [28]. Students with little social experience save for a sense of accomplishment from succeeding in well-structured curriculum with teachers’ full instruction may have only a vague image of becoming a medical professional [38]. This may reflect the difference in professional identity stage between Japanese and U.S. medical students.

Results also reveal the inadequacy of PIF-oriented education at the current preclinical year standard curriculum in Japan. Although the Japanese curriculum is undergoing reform, there are less opportunities for students to be exposed to clinical practice and professionalism education [38–40]. This low base of professional identity stage may have influenced the ineffectiveness of the PIF-oriented intervention, which was administered only three times for each group during the study period.

Response descriptions in the three professional identity essays during the study period provided details of the impact of PIF-oriented intervention on students’ perceptions of their learning (data not shown). According to them, repetitive verbalization of the values and norms of professionalism in PIF-oriented platform helped students perceive responsibilities as a health care professional they had not recognized previously, and felt PBL tasks as realistic. The recognition of a sense of responsibility is reported as a promoter for SRL [16–18, 41, 42]. However, an absolute lack of time for the intervention in this study may have led to the lack of expected results of motivation and learning strategies in the MSLQ.

The PIF-oriented intervention was an attempt to foster SRL within a teacher-centered institutional culture and minimal PBL curriculum. Even though we introduced repeated PIF-oriented pre-PBL platform, we only did so once a month for 2 hours during 8 hours of one-day PBL, while approximately 130 h of didactic lectures per month took place. Therefore, in institutions where teacher-centered culture is prevalent, fostering preclinical student SRL for later clinical clerkship may require more than one short-term intervention.

### Strengths

Subjects in this study were Japanese students placed in a teacher-centered curriculum with teacher-centered institutional culture [16–18]. Therefore, we believe our study can provide educators with evidence that sporadic SRL-oriented educational schemes in such a context cannot improve SRL significantly. Moreover, the experimental setup constitutes an important strength of the study at hand by minimizing threats to internal validity.

### Limitations

JMU is a medical school with a mandatory in-dormitory residence for 6 years, and with a culture in which students frequently share information about their studies in the dormitory. In this study, we permitted only the intervention group students to access the Moodle pre-learning by their IDs and explained in advance that they should not share information with those in the control group. However, some information might have been shared between Groups A and B. A variety of preventive measures can be considered, including investigating at a medical school where students do not board together on campus.

There are no reports that have validated the Japanese version of both MSLQ and PIE. In this study, we used a rigorous back translation by faculty members literate in Japanese and English, but more time examining outcome evaluation methods may be necessary.

## Conclusion

This randomized control study showed no significant effects of PIF-oriented intervention on SRL in a preclinical year curriculum although the repetitive verbalization of the values and norms of professionalism might motivate students by helping them see preclinical PBLs more realistic. Sporadic professional identity formation-oriented intervention conducted outside of clinical settings does not lead to the establishement of SRL in preparation for clinical practice.

## Data Availability

All datasets analyzed during the current study available from the corresponding author on reasonable request.

## Abbreviations

JMU: Jichi Medical University
PBL: Problem-based learning
SRL: Self-regulated learning
PIF: Professional identity formation
PIE: Professional identity essay
PIE-J: Japanese version of PIE
MSLQ: Motivated Strategies for Learning Questionnaire
MSLQ-J: Japanese version of MSLQ
IGO: Intrinsic Goal Orientation
EGO: Extrinsic Goal Orientation
TV: Task Value
CBaL: Control Beliefs about Learning
SEfLaP: Self-Efficacy for Learning and Performance
TA: Test Anxiety
R: Rehearsal
ELA: Elaboration
O: Organization
CT: Critical Thinking
MSR: Metacognitive Self-Regulation
TaSE: Time and Study Environment
ER: Effort Regulation
PL: Peer Learning
HS: Help-Seeking
